# Covariate-adjusted construction of gene regulatory networks using a combination of generalized linear model and penalized maximum likelihood

**DOI:** 10.1371/journal.pone.0309556

**Published:** 2025-01-29

**Authors:** Omid Chatrabgoun, Alireza Daneshkhah, Parisa Torkaman, Mark Johnston, Nader Sohrabi Safa, Ali Kashif Bashir

**Affiliations:** 1 School of Computing, Electronics and Mathematics, Coventry University, Coventry, United Kingdom; 2 Department of Statistics, Faculty of Mathematical Sciences and Statistics, Malayer University, Malayer, Iran; 3 Faculty of Mathematics and Data Science, Emirates Aviation University, Dubai, UAE; 4 Department of Computing and Mathematics, Manchester Metropolitan University, Manchester, United Kingdom; University of Mosul, IRAQ

## Abstract

Many machine learning techniques have been used to construct gene regulatory networks (GRNs) through precision matrix that considers conditional independence among genes, and finally produces sparse version of GRNs. This construction can be improved using the auxiliary information like gene expression profile of the related species or gene markers. To reach out this goal, we apply a generalized linear model (GLM) in first step and later a penalized maximum likelihood to construct the gene regulatory network using Glasso technique for the residuals of a multi-level multivariate GLM among the gene expressions of one species as a multi-levels response variable and the gene expression of related species as a multivariate covariates. By considering the intrinsic property of the gene data which the number of variables is much greater than the number of available samples, a bootstrap version of multi-response multivariate GLM is used. To find most appropriate related species, a cross-validation technique has been used to compute the minimum square error of the fitted GLM under different regularization. The penalized maximum likelihood under a lasso or elastic net penalty is applied on the residual of fitted GLM to find the sparse precision matrix. Finally, we show that the presented algorithm which is a combination of fitted GLM and applying the penalized maximum likelihood on the residual of the model is extremely fast, and can exploit sparsity in the constructed GRNs. Also, we exhibit flexibility of the proposed method presented in this paper by comparing with the other methods to demonstrate the super validity of our approach.

## Introduction

In computer science, network construction techniques find applications in solving bioinformatic challenges, such as the construction of gene regulatory networks (GRNs) from microarray gene expression data, as highlighted by Yang-Yang [1]. This remains a challenging and open problem, mainly due to the high-dimensionality property of microarray data coupled with a limited number of samples, and making it difficult to establish appropriate measures for characterizing gene relationships. Many graphical models have been introduced for gene network construction to infer network edges by utilizing marginal or partial correlations between pairs of genes [2–9]. A standard tool for GRN construction is to fit a Gaussian graphical model to the gene data and then find the empirical sample inverse covariance matrix (precision matrix), and then force certain elements of the precision matrix towards zero [10, 11] to produce a sparse version of GRN construction. However, estimation of the precision matrix using the maximum likelihood (ML) of the fitted Gaussian distribution is reliable only when the fraction (number of variables/number of observations) is very small [12]. Therefore, ML estimation may also lack zero elements and become singular when the number of variables exceeds the number of observations.

Despite the numerous algorithms designed for gene network construction, they can be categorized into partial correlation methods (GeneNet, ENA, SPACE), likelihood methods (CDLasso, Glasso), information theory-based methods (PCA-CMI, CMI2NI), and Bayesian methods (BayesianGlasso). In the partial correlation category, SPACE [13, 19] employs sparse partial correlation estimation, utilizing hub gene information. ENA [14] is an ensemble-based network aggregation method that combines networks from various reconstruction methods into a more accurate single network. GeneNet [15] learns high-dimensional dependency networks from genomic data, allowing users to assign putative directions to edges. In the likelihood methods category, the Coordinate Descent Algorithms for Lasso (CDLasso) [16] is a penalized method using Logistic Regression Coordinate Descent Algorithms for Lasso Penalized Regression. For information theory-based methods, Path Consistency Algorithm based on Conditional Mutual Information (PCA-CMI) [17] infers gene regulatory networks from gene expression data using a path consistency algorithm and conditional mutual information. Conditional Mutual Inclusive Information-based Network Inference (CMI2NI) [18] utilizes a new concept, Conditional Mutual Inclusive Information (CMI2), for accurate measurement of direct dependences between genes. In the Bayesian methods category, Bayesian Graphical Lasso implements a data-augmented block Gibbs sampler for simulating the posterior distribution of concentration matrices. Graphical Lasso (Glasso) [10, 20] is a popular method for estimating a sparse precision matrix, but it exhibits poor behavior in high-dimensional settings like microarray datasets. Extensions and modifications have been made to Glasso, incorporating auxiliary information from other species or knowledge of gene interactions from pathway databases like Pathway Common (PC) and Kyoto Encyclopedia of Genes and Genomes (KEGG). In recent years, the construction of gene regulatory networks (GRNs) has seen significant advancements through the development of various computational algorithms. These algorithms can be broadly categorized based on the underlying methodological approaches they employ. Among these, several prominent methods include the well-conditioned estimator for large-dimensional covariance matrices proposed by Ledoit and Wolf [21]. They introduced an estimator for large-dimensional covariance matrices that is particularly well-conditioned, enhancing the stability and accuracy of network construction. Shahdoust et al. [22] presented the F-MAP method, which incorporates external hints into a Bayesian framework with a Wishart prior to infer GRNs. Additionally, Kuismin and Sillanpää [23] proposed a method utilizing the Wishart prior for sparse precision matrix estimation, offering simple yet effective extensions for network construction.

In this study, a novel approach is proposed for constructing GRNs for one species by leveraging information from related species. This involves a combination of fitting a Generalized Linear Model (GLM) and then applying a penalized maximum likelihood, to enhance the GRN for one species by incorporating auxiliary information from the gene expression profiles of related species. Therefore, what we have done in this paper for the construction of the GRN is summarized in two following steps:

- Step 1: In the first step, we have done multilevel-multivariate GLM under different regularization techniques (Lasso, Ridge, Elastic net) such that the output variable is the gene expression of target specie that we want to construct sparse GRN for that, and the input variable is the best specie that can be used as explanatory variable). We also have fixed the challenge of singularity using bootstrap technique to obtain the residual matrix which is the result of regularized GLM of gene expression of one specie over the gene expression of another specie. To find best specie as a best ancillary information, we have used cross-validation (CV) technique to calculate minimum square error (MSE) criteria as well.
- Step 2: When we obtained the residual matrix from Step 1, we create the penalized likelihood for that matrix by fitting a multivariate Gaussian distribution based on the Glasso technique. And by penalizing this likelihood under different penalties, we obtain the precision matrix. By considering appropriate threshold on this matrix, we constructed sparse GRN for each specie.

The efficiency and sparsity exploitation of the proposed method are demonstrated through a comparison with other methods, highlighting its flexibility and superior validity. The approach is applied to six related species of Drosophila melanogaster, resulting in more accurate precision networks compared to existing methods. The subsequent sections elaborate on a bootstrap version of GLM, utilizing cross-validation to determine proper regularization parameters, followed by results and discussion, and concluding with insights from the findings.

## Materials and methods

Consider a multi-response multivariate GLM with *p* responses and *q* covariates as presented in [24]. Suppose we have *n* independent observations (***x***_*i*_, ***y***_*i*_);*i* = 1, …, *n*, where ***y***_*i*_ = (*y*_*i*1_, …, *y*_*ip*_) and ***x***_*i*_ = (*x*_*i*1_, …, *x*_*iq*_). We assume that the data are centered and standardized. Define ***Y*** = (***y***_1_, …, ***y***_*n*_)^*T*^ and ***X*** = (***x***_1_, …, ***x***_*n*_)^*T*^. In matrix notation,

$$\begin{array}{c} Y_{n\times p}=[\begin{array}{cccc} y_{11} & y_{12} & \cdots & y_{1p}\\ y_{21} & y_{22} & \cdots & y_{2p}\\ \vdots & \vdots & \cdots & \vdots \\ y_{n1} & y_{n2} & \cdots & y_{np} \end{array}]=[\begin{array}{c} y_{1}\\ y_{2}\\ \vdots \\ y_{n} \end{array}], \end{array}$$

we also show the matrix ***X*** by adding the unit vector as its first column as below:

$$\begin{array}{c} X_{n\times (q+1)}=[\begin{array}{c} 1\;\;\\ 1\;\;\\ \vdots \;\;\\ 1\;\; \end{array}\begin{array}{cccc} x_{11} & x_{12} & \cdots & x_{1q}\\ x_{21} & x_{22} & \cdots & x_{2q}\\ \vdots & \vdots & \cdots & \vdots \\ x_{n1} & x_{n2} & \cdots & x_{nq} \end{array}]=[\begin{array}{c} \left(1,x_{1}\right)\\ \left(1,x_{2}\right)\\ \vdots \\ \left(1,x_{n}\right) \end{array}], \end{array}$$

As pointed out in Rencher (2002), the relationship between the multi-level response ***Y*** and the multivariate covariates ***X*** in GLM can be described by the following linear regression model,

$$\begin{array}{c} Y=XB+E, \end{array}$$ (1)

where ***B*** = (***B***_0_, ***B***_1_, …, ***B***_*q*_)^*T*^ is a (*q* + 1) × *p* coefficient matrix such that ***B***_*i*_ = (*β*_*i*1_, *β*_*i*2_, …, *β*_*ip*_) for *i* = 0, 1, …, *q*, and we can write

$$\begin{array}{c} B_{(q+1)\times p}=[\begin{array}{cccc} \beta_{01} & \beta_{02} & \cdots & \beta_{0p}\\ \beta_{11} & \beta_{12} & \cdots & \beta_{1p}\\ \vdots & \vdots & \cdots & \vdots \\ \beta_{q1} & \beta_{q2} & \cdots & \beta_{qp} \end{array}], \end{array}$$ (2)

and ***E*** = (***E***_1_, …, ***E***_*n*_)^*T*^ is the error term such that for *i* = 1, 2, …, *n*, ***E***_*i*_ = (*e*_*i*1_, *e*_*i*2_, …, *e*_*ip*_). We assume that the errors *E*_*i*_ for *i* = 1, …, *n*, are *i*.*i*.*d*. random variables following a multivariate normal distribution N(0,Σ). Let ***y***_*j*_ = (*y*_1*j*_, …, *y*_*nj*_)^*T*^ be the *j*^*th*^ response vector (*j* = 1, .., *p*), based on the multivariate regression of ***y***_*j*_ on ***X*** one can write

$$\begin{array}{c} y_{j}=XB_{j}+E_{j},\;\;\;j=1,2,...,p, \end{array}$$ (3)

where ***B***_*j*_ = (*β*_0*j*_, *β*_1*j*_, …, *β*_*qj*_)^*T*^ and ***E***_*j*_ = (*e*_1*j*_, …, *e*_*nj*_)^*T*^. In a genetic genomics frameworks, ***X*** represents the auxiliary information of similar species and ***Y*** represents the gene expressions of the original species. Our interest is to estimate the coefficient matrix ***B*** = (***B***_0_, ***B***_1_, …, ***B***_*q*_)^*T*^ through *p* multivariate regression (3). By adjusting the effect of ***X*** on ***Y***, conditioning on ***X***, we expect that false positives and/or false negatives of connections in the constructed GRN will be reduced. A traditional approach to estimate ***B***_*j*_ and finally matrix ***B*** is to use ${\hat{B}}_{j}={\left(X^{T}X\right)}^{-1}y_{j},\;\;\;\;j=1,2,...,p,$ Therefore, by estimating ***B***_*j*_, for all *j*, the estimation of coefficient matrix ***B*** can be obtained as below:

$$\begin{array}{c} \begin{array}{cc} \hat{B} & =({\hat{B}}_{1},{\hat{B}}_{2},...,{\hat{B}}_{p})\\ & =({\left(X^{T}X\right)}^{-1}y_{1},{\left(X^{T}X\right)}^{-1}y_{2},...,{\left(X^{T}X\right)}^{-1}y_{p})\\ & ={\left(X^{T}X\right)}^{-1}\left(y_{1},y_{2},...,y_{p}\right)\\ & ={\left(X^{T}X\right)}^{-1}Y, \end{array} \end{array}$$ (4)

We call $\hat{B}$ in (4) the least squares estimator for ***B*** because it minimizes

$$\begin{array}{c} E^{T}E={\left(XB-Y\right)}^{T}\left(XB-Y\right), \end{array}$$

the least squares estimate $\hat{B}$ also minimizes the scalar quantities

$$\begin{array}{c} tr\left[{\left(XB-Y\right)}^{T}\left(XB-Y\right)\right]=\sum_{i=1}^{n}\sum_{j=1}^{p}e_{ij}^{2}, \end{array}$$

Therefore, we implement an estimation procedure with respect to each response in ***Y*** via solving the following *p* optimization problems,

$$\begin{array}{c} {\hat{B}}_{j}=argmin_{B_{j}}tr\left\{{\left(y_{j}-{XB}_{j}\right)}^{T}\left(y_{j}-{XB}_{j}\right)\right\}, \end{array}$$

the complete process of the optimization can be found in [25, 26].

In genetics, the number of variables, i.e. genes, is very high and the number of samples is very small. So, the number of samples is always much smaller than the number of variables. It is often the case that the matrix ***X***^*T*^***X*** is “close” to singular. To overcome this challenge, one can implement a penalized estimation procedure with respect to each response in ***Y*** via solving the following *p* optimization problems,

$$\begin{array}{c} {\hat{B}}_{j}=argmin_{B_{j}}[tr\left\{{\left(y_{j}-{XB}_{j}\right)}^{T}\left(y_{j}-{XB}_{j}\right)\right\}+\lambda_{j}\left|\right|B_{j}{\left|\right|}_{1}], \end{array}$$ (5)

where λ_*j*_, *j* = 1, …, *p*; are tuning parameters, and ||***B***_*j*_||_1_ refers to the *L*_1_-norm (lasso penalty). If the value of λ_*j*_ becomes zero, the model is usual GLM and if its value increases, the number of independent variables in the model will decrease. Therefore, with λ_*j*_ equals to infinite, there will be practically no variables in the model. Determining the value for this parameter is usually performed by the k-fold cross validation (CV) method. For more familiarity with this technique of determing the penalty value, one can refer to Hastie et al. (2015) [27], Efron and Hastie (2016) [28] as well as James et al. (2013) [29]. Hence, our objective is to identify an optimal value, denoted as λ_*j*_, that facilitates the most accurate prediction of response values. It is evident that excessively small λ_*j*_ values may result in overfitting, wherein the model tends to incorporate noise in the data. Conversely, excessively large λ_*j*_ values may lead to underfitting, where the procedure fails to capture the underlying relationship. In both scenarios, a high error value is expected when assessed on the test data, a set of observations not included in the initial sample. To implement the Cross-Validation (CV) technique, we initially partition the original dataset into training and test sets. Subsequently, the training set is utilized to compute the coefficient estimates, which are then validated on the test set. Let’s delve into the algorithm in more detail. The initial dataset is randomly segmented into *Q* blocks of equal length. One of these blocks is designated as the test set, while the remaining *Q* − 1 blocks collectively form the training set. In practice, the number of blocks *Q* (often referred to as k-fold) is typically chosen as 5 or 10. However, by considering the intrinsic property of gene data which the number of variables is much greater than the number of available samples, by dividing training data set into Q blocks in some cases in genetic data for each blocks will remain no more than one or two samples. To fix this issue we apply a bootstrap version of GLM to re-sampling from the existed dataset. Then, the GLM parameters can be computed using CV technique through regularization. We choose a grid search of values $\lambda_{j}=\left[\lambda_{j}^{s}\right]$ and calculate the regression coefficients for each grid $\lambda_{j}^{s}$ value. Given these regression coefficients, we then compute the residual sum of squares (RSS),

$$\begin{array}{c} RSS_{\lambda_{j}^{s}}^{q}=\sum_{i=1}^{n}{\left(y_{i}-\sum_{j=1}^{k}\hat{\beta_{j}}\left(q,\lambda_{j}^{s}\right)x_{ij}\right)}^{2}, \end{array}$$

where $q=\bar{j,Q}$ is the index of the block selected as the test set, and $\hat{\beta_{j}}\left(q,\lambda_{j}^{s}\right)$ stands for the coefficients under the block test set and the grid value of $\lambda_{j}^{s}$. One can obtain the average of these RSS values over all blocks (MSE) as

$$\begin{array}{c} MSE_{\lambda_{j}^{s}}=\frac{1}{Q}\sum_{q=1}^{Q}RSS_{\lambda_{j}^{s}}^{q}, \end{array}$$

where λ_*j*_ is then set equal to $\lambda_{j}^{s}$ that gives the minimum $MSE_{\lambda_{j}^{s}}$. Another popular approach utilizes the “one-standard-error rule” (see for example [30]. For each $MSE_{\lambda_{j}^{s}}$ the standard error of the mean is calculated. Then, we select the largest $\lambda_{j}^{s}$ for which the $MSE_{\lambda_{j}^{s}}$ is within one standard error of the minimum MSE value. This way we obtain a “more regularized” model while increasing the MSE by not more than one standard error. In the following, we apply the LASSO, Ridge and Elastic Net regularization techniques to six species of Drosophila fly. After finding optimal values for λ_*j*_, *j* = 1, …, *p*; we can keep all the MSE values for fitting all GLMs with regularization techniques such that the output variables are the matrix of *Y* (The target spices that we want to construct sparse GRN for that) and the input variables are the matrix of *X* (the best spices that can be used as an explanatory variable), and finally select the best ancillary information for each spices based on the minimum value of the MSE. In fact, we want to construct gene network by fitting a GLM based on the information from the original species that is called the multi-response dependent variable, and the auxiliary information from another species that is called the explanatory multivariate variable. By carry on a GLM between the original species and other species and estimating the coefficients matrix, then the residual matrix for constructing GRNs can be produced. We are going to fit GLM using LASSO, Ridge and Elastic Net regularization. We use the R software for all calculations, namely its *glmnet* package that allows fitting LASSO, Ridge and Elastic Net regularization techniques. When we obtained the residual matrix from the previous step, we create the penalized likelihood for this matrix based on the Glasso technique. And by penalizing this likelihood under different penalties, we obtained the precision matrix. By considering appropriate threshold on this matrix, we constructed sparce GRN for each spice.

In (5), ||.||_1_ refers to *L*_1_ norm means Lasso penalty. It is noteworthy that *L*_2_-norm (Ridge penalty) as well as Elastic net norm, which is a combination of *L*_1_-norm and *L*_2_-norm, can also be used here. However, in the general case, the parameter *α* in the *glmnet* package in R software can be used to create different penalties such that *α* = 1 is applied for Lasso regression and *α* = 0 for Ridge regression. Also, 0 < *α* < 1 is used for the Elastic net norm as follows

$$\begin{array}{c} \left(1-\alpha \right)/{2||B||}_{2}^{2}+{\alpha ||B||}_{1}, \end{array}$$

The advantage of Lasso norm over Ridge norm is that for a large value of λ_*j*_ many of the coefficients of the regression model are precisely zero and this makes it easy to interpret the model.

To assess the effectiveness of our algorithm in network reconstruction, we compute accuracy measures—Precision, Recall, Specificity, and Accuracy—for each network, comparing them to the Gold Standard network.

$$\begin{array}{c} Precision=\frac{TP}{\left(TP+FP\right)},\;\;\;Recall=\frac{TP}{\left(TP+FN\right)}, \end{array}$$

$$\begin{array}{c} Accuracy=\frac{\left(TP+TN\right)}{\left(TP+TN+FN+FP\right)},\;\;\;Specificity=\frac{TN}{\left(TN+FP\right)}. \end{array}$$

Here, *TN* represents the number of true negatives, *FP* is the number of false positives, *FN* is the number of false negatives, and *TP* is the number of true positive edges. For the purpose of comparative analysis, we include both widely adopted and cutting-edge approaches for estimating covariance and precision matrices. Specifically, we consider Ledoit and Wolf [21], Graphical Lasso (Glasso) [10, 31], F-MAP with Wishart prior [22], and the method proposed by Kuismin and SillanpaÈaÈ [23].

## Dataset

**Fig 1:**
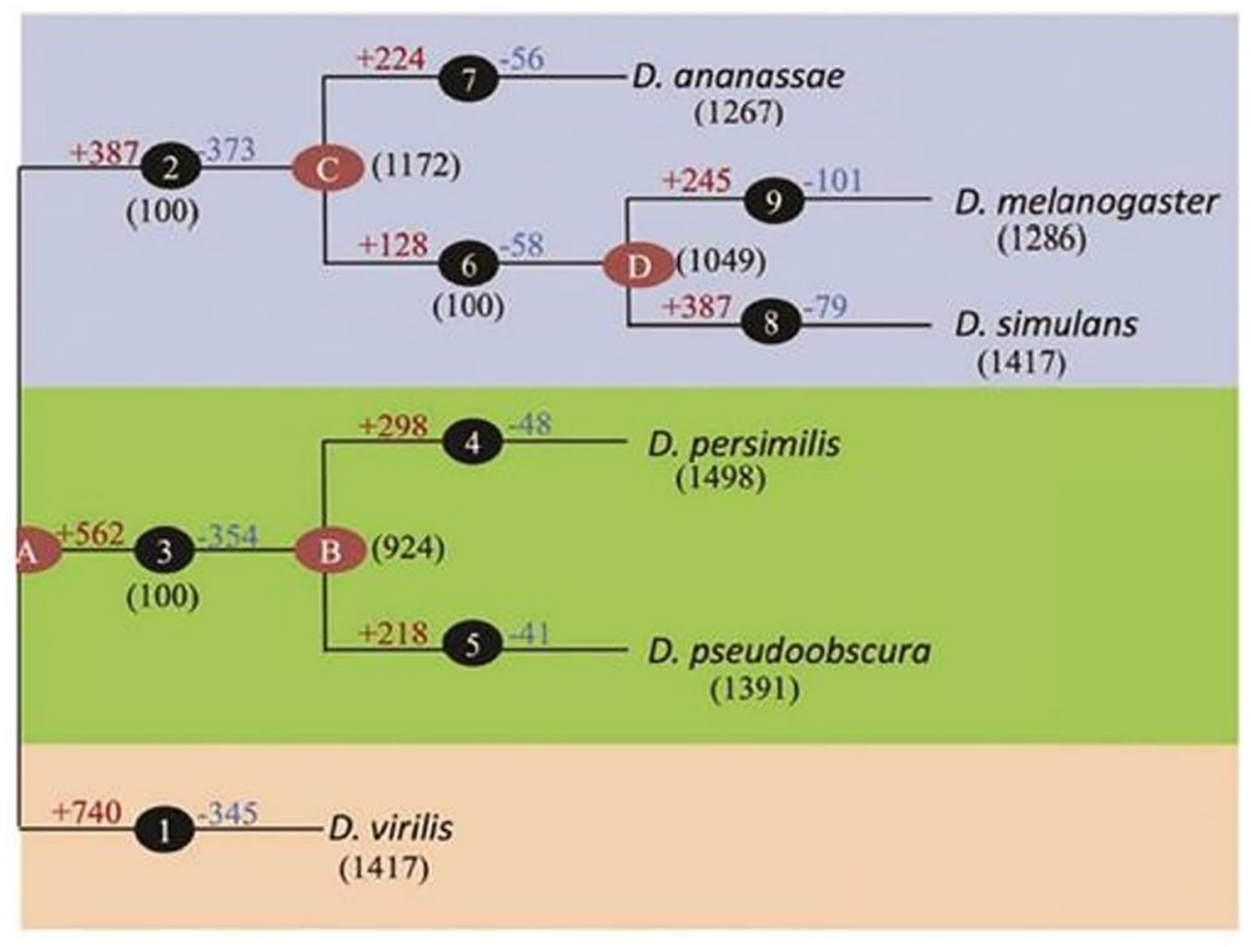
The species’ phylogenetic tree graph is replicated here with the authorization of Joshi et al. (2015).

We applied our methodology to six datasets representing embryonic development time-course expression in Drosophila flies of six different species: D. melanogaster (amel), D. ananassa (ana), D. persimilis (per), D. pseudoobscura (pse), D.simulance (sim), and D.virilis (vir). The phylogenetic tree depicting the relationships among these species is illustrated in Fig 1. The data were sourced from the study by Kalinka et al. [32] and can be accessed in ArrayExpress under the accession code E-MTAB-404. The dataset comprises arrays capturing various developmental time points, with multiple replicates for each species: 10 time points for amel, 13 for vir, and 9 for ana, per, pse, and sim. Gene expressions were processed by averaging over absolute expression levels of different replicates and undergoing a *log*_2_ transformation. We focused on the expression of 2049 genes across the entire dataset, which serve as target genes for twelve transcription factors forming the Gold Standard Network (GSN). A portion of chip-chip data from MacArthur et al. [33] was utilized as the gold standard for gene regulatory networks, encompassing 21 sequence-specific Drosophila transcription factors measured in D. melanogaster embryos. The GSN construction involved considering the relationships between twelve TFs represented on the array and their 2049 target genes, as detailed in Table 1 (TFs and their corresponding target gene counts). Further details about the GSN can be found in [34]. In the subsequent section, we conduct an analysis of the Drosophila fly dataset, aiming to construct Gene Regulatory Networks (GRNs) for each species. This involves fitting a GLM and subsequently applying the Glasso technique to the residuals of the fitted GLM.

**Table 1:** Number of target genes for 12 transcription factors (TFs).

TF	zD	twi	slp1	Sna	run	prd	mad	kr	hb	da	cad	gt1	dl
Number	1166	1164	212	291	158	313	40	518	358	1503	795	273	

## Results

Now, we construct and modify gene regulatory networks (GRNs) for one species using the further information from gene expression profile of the related species as an explanatory variables by performing a multi-level multivariate GLM under different regularizations (Lasso, Ridge and Elasti net). For example, we model the gene expressions of D. melanogaster (*amel*) as a multi-response variable and the gene expression of D. ananassa (*ana*) as a multivariate covariate. Then, we estimate the regression coefficients matrix ***B*** as defined in (1) and (2). At the end, we will find the residual matrix ***E*** in order to construct the adjusted GRNs accordingly by Glasso. Here, we explain how to obtain the results for D. melanogaster (*amel*) and D. ananassa (*ana*), the remaining variables follow the same procedure and only the variables will be changed. For a multi-level response multivariate GLM between D. melanogaster (*amel*) and D. ananassa (*ana*), we have *p* = 10 observations for response variable and also *q* = 9 observations for the covariate such that the number of variables (genes) for both are 2049. We define the relationship between the multi-level response *amel* and the multivariate covariate *ana* by a GLM (1). When we obtain the optimized value of the hyperparameter λ in the regularized GLM using CV techniques with *k* = 10, because the number of variables is much greater than the number of available samples, the model tends to be near singular. Therefore, to solve this problem, we apply a bootstrap version of GLM to re-sample from the existed dataset such that the number of sample for *amel* and *ana* arrived at desirable number of samples. Here, we have arrived at 1000 samples. To choose the number of re-sampling, we have performed an initial experiments to identify a reasonable sample size (e.g., 500 samples), and conduct cross-validation to assess model performance with different sample sizes. Finally, we select a sample size that provides a balance between computational efficiency and model accuracy which was around 1000 samples.

**Fig 2:**
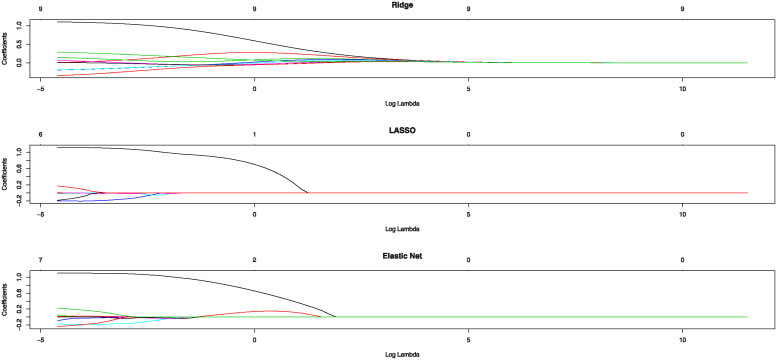
The Coefficient of B_1_ for the LASSO, Ridge and Elastic Net (from top to bottom respectively) versus *log*λ. The upper part of each plot shows the number of non-zero coefficients ***B***_1_ in the fitted GLM.

**Fig 3:**
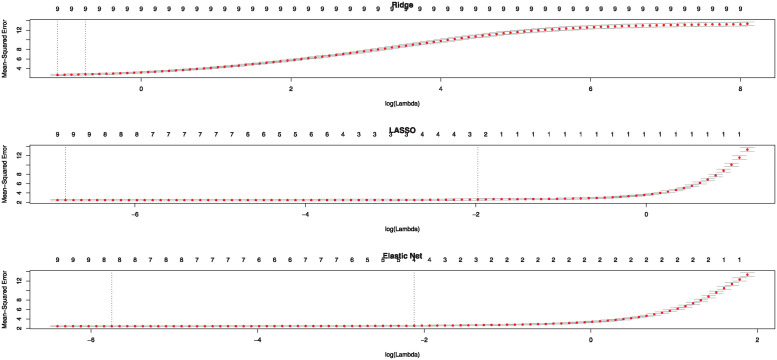
Cross-validated estimate of MSE error for the LASSO, Ridge and Elastic Net penalties (from top to bottom respectively) as a function of *log*λ. The dashed lines show the location of the function minimum and the “one-standard-error” location.

To avoid overlap between re-sampled data, we add random multivariate Gaussian noise with mean vector zero and **Σ** = *σ*^2^ ***I*** with very small *σ*^2^ = 0.001. To select this small value for noise, we start with a small value to introduce optimal noise. And then gradually increase *σ*^2^ and evaluate the impact on model performance that balances noise introduction and model accuracy. Then, we randomly divide the final data set into *Q* = 10 blocks of equal length. One of the blocks is assigned the role of the test set while the remaining 9 blocks together constitute the training set such that for ***Y***_1000×2049_ and ***X***_1000×2050_, we have ***B***_2050×2049_ as coefficient matrix and ***E***_1000×2049_ as the error matrix that should be calculated to construct amended gene network. Note that by using the multivariate regression of ***y***_*j*_ = (*y*_1*j*_, …, *y*_1000×*j*_)^*T*^ that is the *j*th response vector of *amel* on the gene expression of *ana* that is *X*_1000×2050_, we can produce ***B***_*j*_ and ***E***_*j*_ for *j* = 1, .., 2049, the *j*th vector of ***B*** and ***E***, respectively. By repeating this process for all columns of *amel* gene expression (via solving 2049 optimization problems as defined in (5)) the regularized estimation of the coefficient matrix ***B*** ($\hat{B}$) is obtained by fitting 2049 multivariate GLM under *l*_1_-norm or lasso regularization. To find the proper value for the regularization parameter λ, we explain the strategy for the first column of ***Y***_1000×2049_, i.e. ***y***_1_ = (*y*_11_, …, *y*_1000×1_)^*T*^, the remaining 2049 columns follow the same. First, We can visualize the coefficients in ***B_1***, the first column of ***B***, such that the curves in the middle of Fig 2 corresponds to different variables in ***X*** for Lasso regularization. It shows the path of the coefficients in ***B_1*** against the *l*_1_-norm as the regularization parameter λ varies. The axis above indicates the number of nonzero coefficients at the current λ, which is the effective degrees of freedom (df) for the lasso. So, we have so many choices for λ value under lasso penalty. The upper part of the plot shows the number of non-zero coefficients ***B***_1_ in the GLM for a given *log*λ. Also, the Fig 2 shows the coefficient of *B*_1_ for other penalties such that Ridge and Elastic Net regression versus *log*λ. For the lasso, as λ increases, the coefficient estimates ***B***_1_ are “shrunk” towards zero which means that the norm of the estimates vector ||***B***_1_||_2_ decreases. For the Ridge, this number is constant for all the λ values and equals the number of predictors in the data. Thus, although the Ridge regression shrinks the coefficient estimates close to zero, even large λ values do not produce estimates exactly equal to zero. The Elastic net (here 0.5) bridges the gap between lasso and Ridge and mixes them. Therefore, since we have so many choices for λ value under different penalties the Cross-validation (CV) is perhaps the simplest and most widely used method to choose the best one. CV technique returns a list with all the ingredients of the cross-validated fit as is shown in Plot Fig 3. This plot displays the cross-validation curve (depicted by the red dotted line), accompanied by upper and lower standard deviation curves represented as error bars across the λ sequence. Two noteworthy λ values are marked by vertical dotted lines. The λ.*min* corresponds to the λ value associated with the minimum mean cross-validated error, while λ.1*se* corresponds to the λ value that yields the most regularized model, ensuring that the cross-validated error remains within one standard error of the minimum. Therefore, the CV technique is used to find these two proper values of λ.*min*(*bestlam*) and λ.1*se*(*lam*1*se*) for the λ parameter given a sample using (5). For all calculations, the *glmnet* package in R software that allows fitting LASSO, Ridge and Elastic Net GLM has been used. We select the λ parameter using the 10-fold CV procedure such that we utilize the *cv.glmnet* command from the *glmnet* package. In our case, the Ridge regression gives *ridge*.*bestlam* = 0.0036752 and *ridge*.*lam*1*se* = 0.1148763. The LASSO yields *lasso*.*bestlam* = 0.3271718 and *lasso*.*lam*1*se* = 0.4325019 correspondingly. Also, the Elastic Net produces *elastic* − *net*.*bestlam* = 0.004616265 and *elastic* − *net*.*lam*1*se* = 0.1583596 accordingly. Hereafter, we use λ.*min*(*bestlam*) for each fitted GLM between the gene expressions of one species and related species.

Now, we are in a position to find the best related species as an auxiliary information for each species based on the most proper penalty and optimal value of *bestlam*. Then, we evaluate the results based on the MSE measure to find the best multivariate covariate for each species accordingly. Using test set, we are able to measure quality of different GLMs for each species. Here, the *set.seed* command with a fixed input value of 112 is used to get results that can later be reproduced by the reader. As can be seen in Table 2, the best explanatory variable for fitting each GLM for finding the best matrix of residual accordingly is shown as bold number. For example, for *amel* the best explanatory species is *ana* with Elastic Net penalty and the MSE value of 2.99174 that is the minimum value between all other penalties and all other species as multivariate covariate. Therefore, we need to obtain the matrix of residual for models of *amel*∼*ana*, *ana*∼*vir*, *sim*∼*ana*, *per*∼*pse*, *pse*∼*per* and *vir*∼*per*; and then applying Glasso to produce final GRNs.

**Table 2:** Measures of MSE for GLM of Y variables against X variables for λ.*bestlam* and different penalties.

Variables Y∼X		LASSO MSE	Ridge MSE	Elastic Net MSE
amel	ana	3.29001	16.12004	**2.99174**
	sim	**3.7730**	12.34170	3.86851
	per	4.0201	15.84112	**3.90572**
	pse	3.78923	16.55230	**3.73234**
	vir	3.90457	15.02034	**3.88271**
ana	amel	3.992780	17.45931	**3.77229**
	sim	3.938383	20.87798	**3.898258**
	per	3.985274	15.80054	**3.88180**
	pse	3.982974	16.00082	**3.870871**
	vir	3.89924	15.93392	**3.07871**
sim	amel	3.99896	14.74933	**3.64148**
	ana	3.677912	13.45473	**3.634618**
	per	4.105761	16.17947	**3.947643**
	pse	4.01390	14.50563	**3.896814**
	vir	4.00167	14.13239	**3.78279**
per	amel	**3.02094**	13.60881	3.11459
	ana	**3.33179**	12.64797	3.340319
	sim	3.588115	16.30806	**3.569636**
	pse	**2.960796**	10.88034	3.098244
	vir	3.60552	11.73325	**3.21004**
pse	amel	3.809321	14.172185	**3.721294**
	ana	3.626733	12.94426	**3.614632**
	sim	3.954357	16.61443	**3.929095**
	per	**3.204816**	11.02765	3.29077
	vir	**3.336205**	12.178120	3.645420
vir	amel	3.87721	15.452230	**3.301920**
	ana	3.967615	12.145002	**3.851377**
	sim	4.002072	16.524404	**3.90220**
	per	3.794910	12.305780	**3.105564**
	pse	3.778991	11.939420	**3.36041**

Now, after finding the best explanatory variable for each species based on the appropriate penalty and proper value of regularization parameter λ, we want to use Glasso technique; and at the end compare the resulted gene networks for each species with the previous methods based on the different measures of accuracy. When the residual matrix is obtained for each GLM, the final GRNs are constructed using Glasso. For example, we construct GRN for *amel* which is adjusted using ancillary information from *ana*. This means we obtain the covariance matrix for the residual of GLM, and find the precision matrix using Glasso accordingly. To make a sparse precision matrix, the appropriate threshold is set to the 95^th^ percentile of the conditional correlations in the estimated precision matrix. The choice of the 95^th^ percentile as the threshold for the conditional correlations in the estimated precision matrix is based on several considerations aimed at balancing sparsity and retaining significant connections. First, setting a high threshold such as the 95^th^ percentile helps ensure that only the most significant conditional correlations are retained. This approach effectively filters out weaker connections, resulting in a sparser and more interpretable precision matrix. This balance is crucial in high-dimensional data contexts, like gene regulatory networks, where retaining too many weak connections can lead to overfitting and reduced model interpretability. Second, preliminary experiments indicated that thresholds around the 95^th^ percentile provided a good trade-off between model complexity and performance. Lower thresholds tended to retain too many connections, while higher thresholds excessively pruned the network, potentially omitting important interactions. Third, similar approaches have been adopted in related literature, where high-percentile thresholds are used to induce sparsity in precision matrices. While specific percentiles might vary, the principle of using a high percentile to filter significant connections is common.

In Glasso technique, there is a (Non-negative) regularization parameter *ρ*z such that the higher value, the more regularization, the sparser the precision matrix. To choose the optimal value of *ρ*, we have tired the value range of [0.1, 0.2, …, 0.9] to minimize the extended Bayesian Information Criterion (eBIC) proposed in Foygel and others (2010). The diagnostic accuracy measures (TP, Precision, Recall, Accuracy and Specificity) are computed for all methods in comparison with our proposed method in this paper for *amel* in Table 3. Also, the same comparison between proposed method in this paper and other methods in literature for other species are reported in Tables 4 to 8.

**Table 3:** Measures of diagnostic accuracy of constructed networks for amel species.

approach	species	Edges	TP	Precision	Recall	Accuracy	Specificity
Proposed method	ana	897	371	0.45	0.09	0.75	0.96
F-MAP	amel	810	340	0.42	0.05	0.72	0.97
	sim	856	324	0.38	0.05	0.71	0.97
	per	1285	509	0.40	0.07	0.71	0.96
	pse	1036	474	0.46	0.07	0.72	0.97
	vir	1721	609	0.35	0.09	0.70	0.94
Ledoit	-	1635	590	0.36	0.08	0.70	0.94
Kuismin	-	2230	742	0.33	0.11	0.69	0.92
Glasso	-	480	167	0.35	0.02	0.71	0.98

**Table 4:** Measures of diagnostic accuracy of constructed networks for ana species.

approach	species	Edges	TP	Precision	Recall	Accuracy	Specificity
Proposed method	amel	1112	380	0.46	0.09	0.76	0.97
F-MAP	ana	860	393	0.45	0.06	0.72	0.97
	sim	976	472	0.48	0.07	0.72	0.97
	per	1183	517	0.44	0.08	0.72	0.96
	pse	1001	513	0.51	0.07	0.72	0.97
	vir	1604	612	0.38	0.09	0.71	0.94
Ledoit	-	1647	738	0.45	0.11	0.72	0.95
Kuismin	-	1736	758	0.44	0.11	0.71	0.94
Glasso	-	390	207	0.53	0.03	0.72	0.99

**Table 5:** Measures of diagnostic accuracy of constructed networks for sim species.

approach	species	Edges	TP	Precision	Recall	Accuracy	Specificity
Proposed method	ana	946	364	0.45	0.08	0.74	0.97
F-MAP	amel	802	349	0.43	0.05	0.72	0.97
	ana	819	303	0.37	0.04	0.71	0.97
	per	1246	478	0.38	0.07	0.71	0.96
	pse	984	445	0.45	0.06	0.72	0.97
	vir	1739	633	0.36	0.09	0.70	0.94
Ledoit	-	1550	574	0.37	0.08	0.70	0.94
Kuismin	-	2461	968	0.39	0.14	0.70	0.90
Glasso	-	619	274	0.44	0.04	0.72	0.98

**Table 6:** Measures of diagnostic accuracy of constructed networks for per species.

approach	species	Edges	TP	Precision	Recall	Accuracy	Specificity
Proposed method	pse	1556	780	0.46	0.11	0.76	0.96
F-MAP	amel	1595	710	0.45	0.10	0.72	0.95
	sim	1556	707	0.45	0.10	0.72	0.95
	amel	1438	678	0.47	0.10	0.72	0.96
	pse	1761	823	0.47	0.11	0.72	0.95
	vir	2014	791	0.39	0.11	0.71	0.93
Ledoit	-	2389	994	0.42	0.14	0.71	0.92
Kuismin	-	1980	770	0.39	0.11	0.70	0.93
Glasso	-	423	179	0.42	0.03	0.72	0.99

**Table 7:** Measures of diagnostic accuracy of constructed networks for pse species.

approach	species	Edges	TP	Precision	Recall	Accuracy	Specificity
Proposed method	per	1457	676	0.48	0.11	0.76	0.96
F-MAP	ana	1318	624	0.47	0.09	0.72	0.96
	sim	1304	600	0.46	0.09	0.72	0.96
	per	1608	696	0.43	0.10	0.71	0.95
	amel	1162	590	0.05	0.08	0.72	0.97
	vir	1959	793	0.40	0.11	0.71	0.93
Ledoit	-	2143	932	0.43	0.13	0.71	0.93
Kuismin	-	1859	600	0.45	0.14	0.70	0.93
Glasso	-	432	186	0.43	0.02	0.72	0.99

**Table 8:** Measures of diagnostic accuracy of constructed networks for vir species.

approach	species	Edges	TP	Precision	Recall	Accuracy	Specificity
Proposed method	per	2142	942	0.48	0.14	0.76	0.95
F-MAP	ana	1951	890	0.46	0.13	0.72	0.94
	sim	2031	915	0.45	0.13	0.71	0.94
	per	2094	964	0.46	0.14	0.71	0.94
	pse	2117	996	0.47	0.15	0.72	0.94
	amel	1881	873	0.46	0.13	0.72	0.94
Ledoit	-	2622	1181	0.45	0.17	0.71	0.92
Kuismin	-	2138	976	0.46	0.14	0.72	0.93
Glasso	-	246	144	0.58	0.02	0.72	0.99

**Fig 4:**
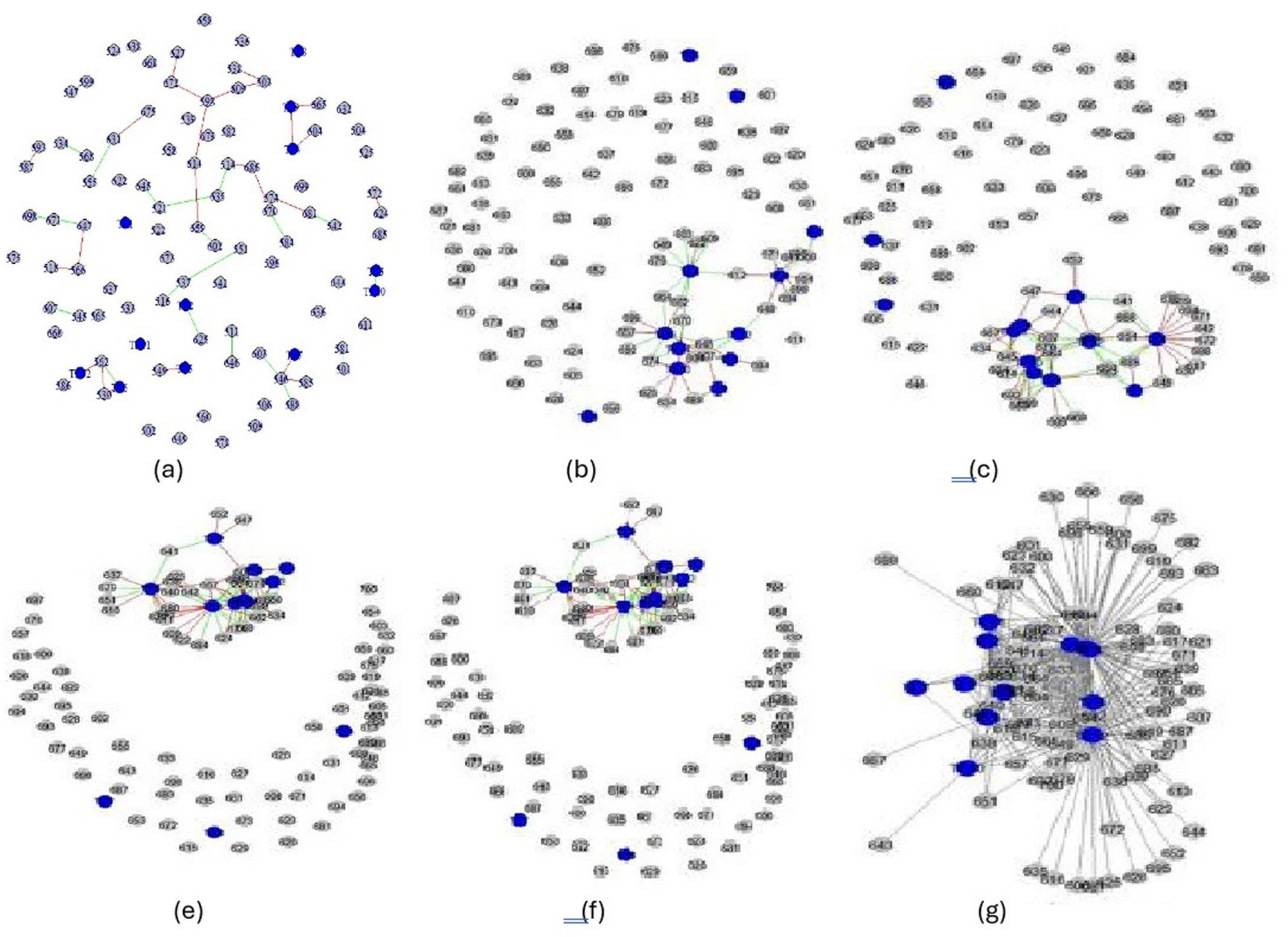
Sub-networks (interactions among 100 genes) for amel presented for different methods: (a) Proposed method in this paper for ame, (b) F-MAP [22], (c) Ledoit & Wolf [21], (d) Kuismin & Sillanpaa [23], (e) GLasso [10, 31]and (f) Gold Standard.

According to the obtained results from the proposed method in this paper, we plot final GRN for amel that is adjusted based on the auxiliary information from ana. We have truncated the final network for other species for brevity. Since the constructed networks include the large number of edges, we just illustrate some parts of the final networks for amel Fig 4. These graphs represent the interactions between 12 TFs and 100 genes. To choose these genes, we partitioned the gene set to 21 groups and chose the one at random. The blue and grey nodes indicate the TFs and their target genes, respectively. The red and green lines indicate the false and true edges, respectively. All five graphs are sparser than the gold standard one.

## Discussion

We systematically interpret and evaluate the results obtained for each species individually, meticulously comparing the final outcomes of the method proposed in this paper with those of previous methods. This comparative analysis is conducted based on various measures of accuracy to ensure a comprehensive assessment of our approach’s performance.

**amel:** Proposed method in this paper has the highest accuracy (75%) in comparison with all other methods. The Precision value is higher than all previous methods except F-MAP’s method when pse has been used as an auxiliary information. The specificity of our method is 96%, so it is very close to the other methods. Recall is 0.08% that is smaller than Kuismin’s method and mostly equals or higher than all other methods. So, the performance of our method is appropriate.

**ana:** The proposed method presented in this paper demonstrates the highest accuracy rate of 76% when compared to all other methods. In terms of Precision, our method outperforms all previous approaches except for F-MAP’s method when pse is utilized as auxiliary information. With a specificity of 97%, our method closely aligns with the performance of other methods. However, the recall rate of our method, at 0.09%, is lower than that of Ledoit’s method and Kuismin’s method, but generally comparable to or higher than other methods. Therefore, based on these findings, we can conclude that our method exhibits a satisfactory level of performance.

**sim:** The proposed method in this paper achieves the highest accuracy rate of 74% compared to all other methods examined. Additionally, the Precision value of our method surpasses that of all previous methods. With a specificity of 97%, our method closely aligns with the performance of the other methods evaluated. However, the recall rate of our method, at 0.08%, is lower than that of Kuismin’s method, while generally being equal to or higher than the recall rates of other methods.

**per:** The proposed method presented in this paper achieves the highest accuracy rate of 76% compared to all other methods investigated. Moreover, the Precision value of our method surpasses that of all previous methods, except for F-MAP’s method when amel and pse are employed as auxiliary information. In terms of specificity, our method exhibits a value of 96%, which closely aligns with the performance of the other methods. However, the recall rate of our method, at 0.09%, is lower than that of Ledoit’s method, yet generally comparable to or higher than other methods. Based on these results, we can conclude that the performance of our method is deemed appropriate.

**pse:** The proposed method described in this paper achieves the highest accuracy rate of 76% when compared to all other methods examined. Furthermore, the Precision value of our method surpasses that of all previous methods, except for F-MAP’s method when pse is utilized as auxiliary information. With a specificity of 97%, our method closely aligns with the performance of the other methods investigated. However, the recall rate of our method, at 0.11%, is lower than that of Ledoit’s method, yet generally comparable to or higher than other methods. Therefore, based on these findings, we can conclude that the performance of our method is considered appropriate.

**vir:** The proposed method presented in this paper demonstrates the highest accuracy rate of 76% compared to all other methods analyzed. Additionally, the Precision value of our method surpasses that of all previous methods, except for the Glasso method. With a specificity of 95%, our method closely aligns with the performance of the other methods assessed. However, the recall rate of our method, at 0.14%, is lower than that of Ledoit’s method, yet generally comparable to or higher than other methods. Based on these results, we can conclude that the performance of our method is considered appropriate.

Therefore, these measures strongly support the notion that our method significantly enhances the quality of the constructed networks when compared to three other approaches.

## Conclusion

In this paper we introduced ML technique to construct gene regulatory networks (GRNs) through precision matrix that considers conditional independence between genes. This GRN construction was improved using the external knowledge about gene interactions drawn from the other related species gene expression data. We used a combination of fitted GLM and penalized maximum likelihood to construct GRN based on the residuals of a multi-level multivariate GLM between the gene expressions of one species as a multi-levels response variable and the gene expression of related species as a multivariate covariates. By considering the intrinsic property of gene data which the number of variables is much greater than the number of available samples, a bootstrap version of multi-response multivariate GLM was computed that uses cross-validation to find the regularization parameters. Then, we constructed GRNs based on the obtained covariance matrix produced from the residual of the fitted GLM using final algorithm of adjusted Glasso. Finally, we showed that the presented algorithm is extremely is accurate. Also, we exhibited flexibility of the proposed method presented in this paper by comparing with the other methods to demonstrate the super validity of our approach.

## Supporting information

S1 Fig The species’ phylogenetic tree graph is replicated here with the authorization of Joshi et al. (2015). (JPG)

S2 Fig Coefficient of B_1_ for the LASSO, Ridge and Elastic Net (from top to bottom respectively) versus *log*λ. The upper part of each plot shows the number of non-zero coefficients B_1_ in the fitted GLM.
(EPS)

S3 Fig Cross-validated estimate of MSE error for the LASSO, Ridge and Elastic Net penalties (from top to bottom respectively) as a function of *log*λ. The dashed lines show the location of the function minimum and the “one-standard-error” location.
(EPS)

S4 Fig Sub-networks (interactions among 100 genes) for amel presented for different methods. (JPG)

S1 File Number of target genes for 12 transcription factors (TFs). (PDF)

S2 File Measures of MSE for GLM of *Y* variables against *X* variables for λ.bestlam and different penalties. (PDF)

S3 File Measures of diagnostic accuracy of constructed networks for amel species. (PDF)

S4 File Measures of diagnostic accuracy of constructed networks for ana species. (PDF)

S5 File Measures of diagnostic accuracy of constructed networks for sim species. (PDF)

S6 File Measures of diagnostic accuracy of constructed networks for per species. (PDF)

S7 File Measures of diagnostic accuracy of constructed networks for pse species. (PDF)

S8 File Measures of diagnostic accuracy of constructed networks for vir species. (PDF)
