# Supplementary figures and images for: Covariate-adjusted construction of gene regulatory networks using a combination of generalized linear model and penalized maximum likelihood
Source: PLoS One. 2025 Jan 29;20(1):e0309556. doi: 10.1371/journal.pone.0309556 (PMC11778759; doi:10.1371/journal.pone.0309556)

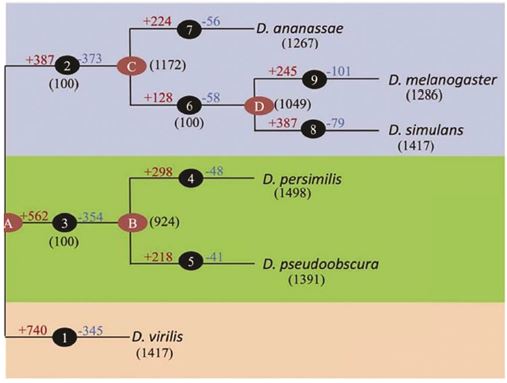

Supplement: S1 Fig — (JPG) [file pone.0309556.s001.jpg]

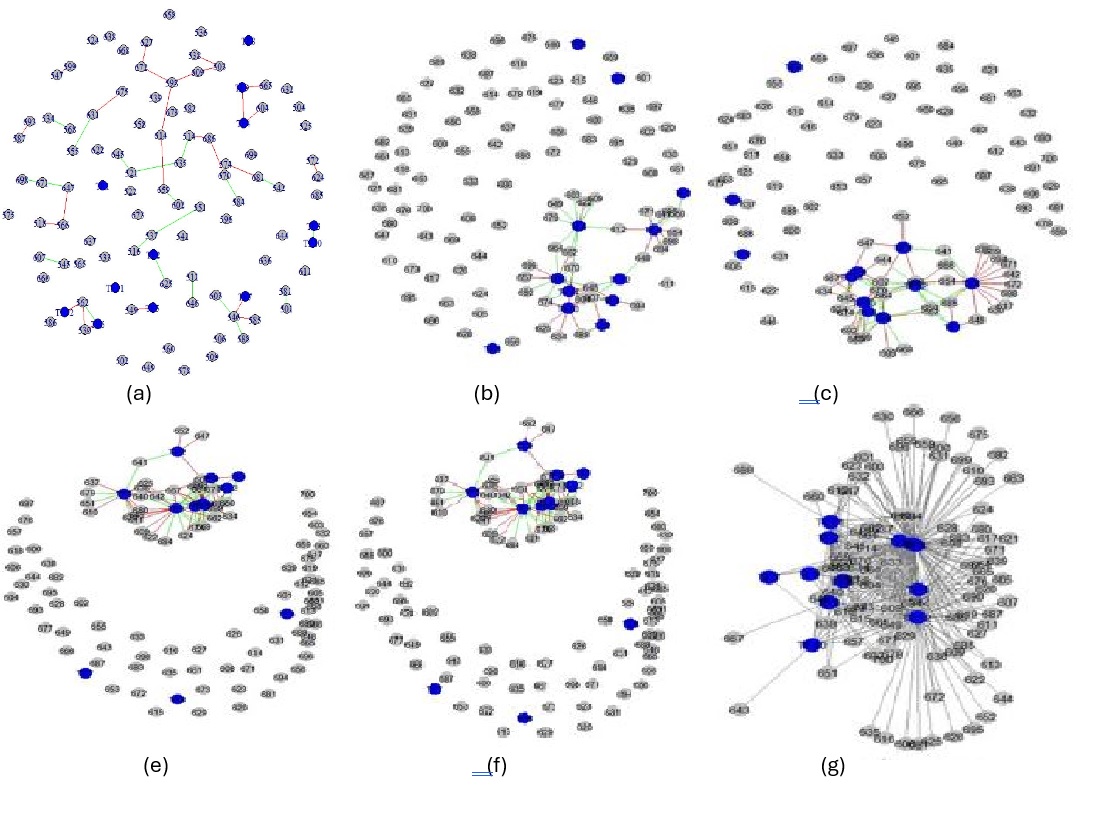

Supplement: S4 Fig — (JPG) [file pone.0309556.s004.jpg]
