# Supplementary material for: Covariate-adjusted construction of gene regulatory networks using a combination of generalized linear model and penalized maximum likelihood
Source: PLoS One. 2025 Jan 29;20(1):e0309556. doi: 10.1371/journal.pone.0309556 (PMC11778759; doi:10.1371/journal.pone.0309556)
Supplement: S1 File — (PDF) [file pone.0309556.s005.pdf]

Table 1. Number of target genes for 12 transcription factors (TFs).

TF	zD	twi	slp1	Sna	run	prd	mad	kr	hb	da	cad	gt1 dl
Number	1166	1164	212	291	158	313	40	518	358	1503	795	273

Table 2. Measures of MSE for GLM of Y variables against X variables for $\lambda.bestlam$ and different penalties.

Variables Y~X		LASSO MSE	Ridge MSE	Elastic Net MSE
amel	ana	3.29001	16.12004	**2.99174**
	sim	**3.7730**	12.34170	3.86851
	per	4.0201	15.84112	**3.90572**
	pse	3.78923	16.55230	**3.73234**
	vir	3.90457	15.02034	**3.88271**
ana	amel	3.992780	17.45931	**3.77229**
	sim	3.938383	20.87798	**3.898258**
	per	3.985274	15.80054	**3.88180**
	pse	3.982974	16.00082	**3.870871**
	vir	3.89924	15.93392	**3.07871**
sim	amel	3.99896	14.74933	**3.64148**
	ana	3.677912	13.45473	**3.634618**
	per	4.105761	16.17947	**3.947643**
	pse	4.01390	14.50563	**3.896814**
	vir	4.00167	14.13239	**3.78279**
per	amel	**3.02094**	13.60881	3.11459
	ana	**3.33179**	12.64797	3.340319
	sim	3.588115	16.30806	**3.569636**
	pse	**2.960796**	10.88034	3.098244
	vir	3.60552	11.73325	**3.21004**
pse	amel	3.809321	14.172185	**3.721294**
	ana	3.626733	12.94426	**3.614632**
	sim	3.954357	16.61443	**3.929095**
	per	**3.204816**	11.02765	3.29077
	vir	**3.336205**	12.178120	3.645420
vir	amel	3.87721	15.452230	**3.301920**
	ana	3.967615	12.145002	**3.851377**
	sim	4.002072	16.524404	**3.90220**
	per	3.794910	12.305780	**3.105564**
	pse	3.778991	11.939420	**3.36041**

Table 3. Measures of diagnostic accuracy of constructed networks for amel species

approach	species	Edges	TP	Precision	Recall	Accuracy	Specificity
Proposed method	ana	897	371	0.45	0.09	0.75	0.96
F-MAP	amel	810	340	0.42	0.05	0.72	0.97
	sim	856	324	0.38	0.05	0.71	0.97
	per	1285	509	0.40	0.07	0.71	0.96
	pse	1036	474	0.46	0.07	0.72	0.97
	vir	1721	609	0.35	0.09	0.70	0.94
Ledoit	-	1635	590	0.36	0.08	0.70	0.94
Kuismin	-	2230	742	0.33	0.11	0.69	0.92
Glasso	-	480	167	0.35	0.02	0.71	0.98

Table 4. Measures of diagnostic accuracy of constructed networks for ana species.

approach	species	Edges	TP	Precision	Recall	Accuracy	Specificity
Proposed method	amel	1112	380	0.46	0.09	0.76	0.97
F-MAP	ana	860	393	0.45	0.06	0.72	0.97
	sim	976	472	0.48	0.07	0.72	0.97
	per	1183	517	0.44	0.08	0.72	0.96
	pse	1001	513	0.51	0.07	0.72	0.97
	vir	1604	612	0.38	0.09	0.71	0.94
Ledoit	-	1647	738	0.45	0.11	0.72	0.95
Kuismin	-	1736	758	0.44	0.11	0.71	0.94
Glasso	-	390	207	0.53	0.03	0.72	0.99

Table 5. Measures of diagnostic accuracy of constructed networks for sim species.

approach	species	Edges	TP	Precision	Recall	Accuracy	Specificity
Proposed method	ana	946	364	0.45	0.08	0.74	0.97
F-MAP	amel	802	349	0.43	0.05	0.72	0.97
	ana	819	303	0.37	0.04	0.71	0.97
	per	1246	478	0.38	0.07	0.71	0.96
	pse	984	445	0.45	0.06	0.72	0.97
	vir	1739	633	0.36	0.09	0.70	0.94
Ledoit	-	1550	574	0.37	0.08	0.70	0.94
Kuismin	-	2461	968	0.39	0.14	0.70	0.90
Glasso	-	619	274	0.44	0.04	0.72	0.98

Table 6. Measures of diagnostic accuracy of constructed networks for per species.

approach	species	Edges	TP	Precision	Recall	Accuracy	Specificity
Proposed method	pse	1556	780	0.46	0.11	0.76	0.96
F-MAP	amel	1595	710	0.45	0.10	0.72	0.95
	sim	1556	707	0.45	0.10	0.72	0.95
	amel	1438	678	0.47	0.10	0.72	0.96
	pse	1761	823	0.47	0.11	0.72	0.95
	vir	2014	791	0.39	0.11	0.71	0.93
Ledoit	-	2389	994	0.42	0.14	0.71	0.92
Kuismin	-	1980	770	0.39	0.11	0.70	0.93
Glasso	-	423	179	0.42	0.03	0.72	0.99

Table 7. Measures of diagnostic accuracy of constructed networks for pse species.

approach	species	Edges	TP	Precision	Recall	Accuracy	Specificity
Proposed method	per	1457	676	0.48	0.11	0.76	0.96
F-MAP	ana	1318	624	0.47	0.09	0.72	0.96
	sim	1304	600	0.46	0.09	0.72	0.96
	per	1608	696	0.43	0.10	0.71	0.95
	amel	1162	590	0.05	0.08	0.72	0.97
	vir	1959	793	0.40	0.11	0.71	0.93
Ledoit	-	2143	932	0.43	0.13	0.71	0.93
Kuismin	-	1859	600	0.45	0.14	0.70	0.93
Glasso	-	432	186	0.43	0.02	0.72	0.99

Table 8. Measures of diagnostic accuracy of constructed networks for vir species.

approach	species	Edges	TP	Precision	Recall	Accuracy	Specificity
Proposed method	per	2142	942	0.48	0.14	0.76	0.95
F-MAP	ana	1951	890	0.46	0.13	0.72	0.94
	sim	2031	915	0.45	0.13	0.71	0.94
	per	2094	964	0.46	0.14	0.71	0.94
	pse	2117	996	0.47	0.15	0.72	0.94
	amel	1881	873	0.46	0.13	0.72	0.94
Ledoit	-	2622	1181	0.45	0.17	0.71	0.92
Kuismin	-	2138	976	0.46	0.14	0.72	0.93
Glasso	-	246	144	0.58	0.02	0.72	0.99
