# Supplementary material for: Covariate-adjusted construction of gene regulatory networks using a combination of generalized linear model and penalized maximum likelihood
Source: PLoS One. 2025 Jan 29;20(1):e0309556. doi: 10.1371/journal.pone.0309556 (PMC11778759; doi:10.1371/journal.pone.0309556)
Supplement: S2 File — (PDF) [file pone.0309556.s006.pdf]

**Table 2.** Measures of MSE for GLM of Y variables against X variables for $\lambda.bestlam$ and different penalties.

| Variables Y~X | | LASSO MSE | Ridge MSE | Elastic Net MSE |
|---|---|---|---|---|
| amel | ana | 3.29001 | 16.12004 | **2.99174** |
| | sim | **3.7730** | 12.34170 | 3.86851 |
| | per | 4.0201 | 15.84112 | **3.90572** |
| | pse | 3.78923 | 16.55230 | **3.73234** |
| | vir | 3.90457 | 15.02034 | **3.88271** |
| ana | amel | 3.992780 | 17.45931 | **3.77229** |
| | sim | 3.938383 | 20.87798 | **3.898258** |
| | per | 3.985274 | 15.80054 | **3.88180** |
| | pse | 3.982974 | 16.00082 | **3.870871** |
| | vir | 3.89924 | 15.93392 | **3.07871** |
| sim | amel | 3.99896 | 14.74933 | **3.64148** |
| | ana | 3.677912 | 13.45473 | **3.634618** |
| | per | 4.105761 | 16.17947 | **3.947643** |
| | pse | 4.01390 | 14.50563 | **3.896814** |
| | vir | 4.00167 | 14.13239 | **3.78279** |
| per | amel | **3.02094** | 13.60881 | 3.11459 |
| | ana | **3.33179** | 12.64797 | 3.340319 |
| | sim | 3.588115 | 16.30806 | **3.569636** |
| | pse | **2.960796** | 10.88034 | 3.098244 |
| | vir | 3.60552 | 11.73325 | **3.21004** |
| pse | amel | 3.809321 | 14.172185 | **3.721294** |
| | ana | 3.626733 | 12.94426 | **3.614632** |
| | sim | 3.954357 | 16.61443 | **3.929095** |
| | per | **3.204816** | 11.02765 | 3.29077 |
| | vir | **3.336205** | 12.178120 | 3.645420 |
| vir | amel | 3.87721 | 15.452230 | **3.301920** |
| | ana | 3.967615 | 12.145002 | **3.851377** |
| | sim | 4.002072 | 16.524404 | **3.90220** |
| | per | 3.794910 | 12.305780 | **3.105564** |
| | pse | 3.778991 | 11.939420 | **3.36041** |
