# Supplementary material for: Covariate-adjusted construction of gene regulatory networks using a combination of generalized linear model and penalized maximum likelihood
Source: PLoS One. 2025 Jan 29;20(1):e0309556. doi: 10.1371/journal.pone.0309556 (PMC11778759; doi:10.1371/journal.pone.0309556)
Supplement: S3 File — (PDF) [file pone.0309556.s007.pdf]

Table 3. Measures of diagnostic accuracy of constructed networks for amel species

approach	species	Edges	TP	Precision	Recall	Accuracy	Specificity
Proposed method	ana	897	371	0.45	0.09	0.75	0.96
F-MAP	amel	810	340	0.42	0.05	0.72	0.97
	sim	856	324	0.38	0.05	0.71	0.97
	per	1285	509	0.40	0.07	0.71	0.96
	pse	1036	474	0.46	0.07	0.72	0.97
	vir	1721	609	0.35	0.09	0.70	0.94
Ledoit	-	1635	590	0.36	0.08	0.70	0.94
Kuismin	-	2230	742	0.33	0.11	0.69	0.92
Glasso	-	480	167	0.35	0.02	0.71	0.98
