# Supplementary material for: Covariate-adjusted construction of gene regulatory networks using a combination of generalized linear model and penalized maximum likelihood
Source: PLoS One. 2025 Jan 29;20(1):e0309556. doi: 10.1371/journal.pone.0309556 (PMC11778759; doi:10.1371/journal.pone.0309556)
Supplement: S4 File — (PDF) [file pone.0309556.s008.pdf]

Table 4. Measures of diagnostic accuracy of constructed networks for ana species.

approach	species	Edges	TP	Precision	Recall	Accuracy	Specificity
Proposed method	amel	1112	380	0.46	0.09	0.76	0.97
F-MAP	ana	860	393	0.45	0.06	0.72	0.97
	sim	976	472	0.48	0.07	0.72	0.97
	per	1183	517	0.44	0.08	0.72	0.96
	pse	1001	513	0.51	0.07	0.72	0.97
	vir	1604	612	0.38	0.09	0.71	0.94
Ledoit	-	1647	738	0.45	0.11	0.72	0.95
Kuismin	-	1736	758	0.44	0.11	0.71	0.94
Glasso	-	390	207	0.53	0.03	0.72	0.99
