# Supplementary material for: Covariate-adjusted construction of gene regulatory networks using a combination of generalized linear model and penalized maximum likelihood
Source: PLoS One. 2025 Jan 29;20(1):e0309556. doi: 10.1371/journal.pone.0309556 (PMC11778759; doi:10.1371/journal.pone.0309556)
Supplement: S5 File — (PDF) [file pone.0309556.s009.pdf]

Table 5. Measures of diagnostic accuracy of constructed networks for sim species.

approach	species	Edges	TP	Precision	Recall	Accuracy	Specificity
Proposed method	ana	946	364	0.45	0.08	0.74	0.97
F-MAP	amel	802	349	0.43	0.05	0.72	0.97
	ana	819	303	0.37	0.04	0.71	0.97
	per	1246	478	0.38	0.07	0.71	0.96
	pse	984	445	0.45	0.06	0.72	0.97
	vir	1739	633	0.36	0.09	0.70	0.94
Ledoit	-	1550	574	0.37	0.08	0.70	0.94
Kuismin	-	2461	968	0.39	0.14	0.70	0.90
Glasso	-	619	274	0.44	0.04	0.72	0.98
