# Supplementary material for: Covariate-adjusted construction of gene regulatory networks using a combination of generalized linear model and penalized maximum likelihood
Source: PLoS One. 2025 Jan 29;20(1):e0309556. doi: 10.1371/journal.pone.0309556 (PMC11778759; doi:10.1371/journal.pone.0309556)
Supplement: S6 File — (PDF) [file pone.0309556.s010.pdf]

Table 6. Measures of diagnostic accuracy of constructed networks for per species.

approach	species	Edges	TP	Precision	Recall	Accuracy	Specificity
Proposed method	pse	1556	780	0.46	0.11	0.76	0.96
F-MAP	amel	1595	710	0.45	0.10	0.72	0.95
	sim	1556	707	0.45	0.10	0.72	0.95
	amel	1438	678	0.47	0.10	0.72	0.96
	pse	1761	823	0.47	0.11	0.72	0.95
	vir	2014	791	0.39	0.11	0.71	0.93
Ledoit	-	2389	994	0.42	0.14	0.71	0.92
Kuismin	-	1980	770	0.39	0.11	0.70	0.93
Glasso	-	423	179	0.42	0.03	0.72	0.99
