# Supplementary material for: Covariate-adjusted construction of gene regulatory networks using a combination of generalized linear model and penalized maximum likelihood
Source: PLoS One. 2025 Jan 29;20(1):e0309556. doi: 10.1371/journal.pone.0309556 (PMC11778759; doi:10.1371/journal.pone.0309556)
Supplement: S7 File — (PDF) [file pone.0309556.s011.pdf]

Table 7. Measures of diagnostic accuracy of constructed networks for pse species.

approach	species	Edges	TP	Precision	Recall	Accuracy	Specificity
Proposed method	per	1457	676	0.48	0.11	0.76	0.96
F-MAP	ana	1318	624	0.47	0.09	0.72	0.96
	sim	1304	600	0.46	0.09	0.72	0.96
	per	1608	696	0.43	0.10	0.71	0.95
	amel	1162	590	0.05	0.08	0.72	0.97
	vir	1959	793	0.40	0.11	0.71	0.93
Ledoit	-	2143	932	0.43	0.13	0.71	0.93
Kuismin	-	1859	600	0.45	0.14	0.70	0.93
Glasso	-	432	186	0.43	0.02	0.72	0.99
