# Supplementary material for: Covariate-adjusted construction of gene regulatory networks using a combination of generalized linear model and penalized maximum likelihood
Source: PLoS One. 2025 Jan 29;20(1):e0309556. doi: 10.1371/journal.pone.0309556 (PMC11778759; doi:10.1371/journal.pone.0309556)
Supplement: S8 File — (PDF) [file pone.0309556.s012.pdf]

Table 8. Measures of diagnostic accuracy of constructed networks for vir species.

approach	species	Edges	TP	Precision	Recall	Accuracy	Specificity
Proposed method	per	2142	942	0.48	0.14	0.76	0.95
F-MAP	ana	1951	890	0.46	0.13	0.72	0.94
	sim	2031	915	0.45	0.13	0.71	0.94
	per	2094	964	0.46	0.14	0.71	0.94
	pse	2117	996	0.47	0.15	0.72	0.94
	amel	1881	873	0.46	0.13	0.72	0.94
Ledoit	-	2622	1181	0.45	0.17	0.71	0.92
Kuismin	-	2138	976	0.46	0.14	0.72	0.93
Glasso	-	246	144	0.58	0.02	0.72	0.99
